# Heme Oxygenase-1 in Bone Remodeling: Molecular Mechanisms and Therapeutic Implications

**DOI:** 10.3390/biom16091224

**Published:** 2026-08-23

**Authors:** Thanawat Pattananandecha, Sutasinee Apichai, Chalermpong Saenjum, Young-Joon Surh

**Affiliations:** 1 Office of Research Administration, Chiang Mai University, Chiang Mai 50200, Thailand; thanawat.pdecha@gmail.com (T.P.); sutasinee.apichai@gmail.com (S.A.); 2 Research Center for Innovation in Analytical Science and Technology for Biodiversity-Based Economic and Society (I-ANALY-S-T_B.BES-CMU), Chiang Mai University, Chiang Mai 50200, Thailand; 3 Department of Pharmaceutical Sciences, Faculty of Pharmacy, Chiang Mai University, Chiang Mai 50200, Thailand; 4 College of Pharmacy, Seoul National University, Seoul 08826, Republic of Korea

**Keywords:** heme oxygenase-1, bone remodeling, osteoblast differentiation, osteoclastogenesis, RANK-RANKL-osteoprotegerin axis

## Abstract

Bone remodeling is a dynamic and tightly regulated process that maintains skeletal homeostasis through a balance between bone formation by osteoblasts and bone resorption by osteoclasts. Disruption of this balance contributes to the development of bone-related disorders, particularly osteopenia, osteoporosis and osteogenesis imperfecta, which weaken, deform, or cause fractures. Increasing evidence indicates that oxidative stress and chronic inflammation impair osteoblast functions while promoting osteoclast differentiation and activity. Heme oxygenase-1 (HO-1) is a stress-inducible enzyme with cytoprotective, antioxidant, and anti-inflammatory properties. Besides its primary role in cellular defense against oxidative stress and inflammatory damage, HO-1 has been shown to be involved in both osteoblast differentiation and osteoclastogenesis. Through its interaction with key regulatory systems, including the receptor activator of nuclear factor κB (RANK)–receptor activator of nuclear factor κB ligand (RANKL)–osteoprotegerin axis and redox-sensitive signaling pathways, HO-1 contributes to maintenance of optimal bone remodeling. The enzyme also plays a role in modulating metabolic processes in the bone. This review highlights the role of HO-1 in bone formation, bone resorption, and related pathophysiologic conditions. Furthermore, the therapeutic potential of HO-1 as a target for bone disorders is discussed.

## 1. Introduction

Bone is a dynamic tissue with important mechanical and metabolic functions. It undergoes continuous remodeling, maintaining a balance between bone resorption by osteoclasts derived from the hematopoietic lineage and bone formation by osteoblasts derived from the mesenchymal lineage [1,2]. Osteoblasts and osteoclasts are functionally coupled during bone remodeling, with reciprocal signals coordinating bone formation and bone resorption. Osteoblast-derived osteoprotegerin (OPG) and receptor activator of nuclear factor κB ligand (RANKL) regulate osteoclastogenesis, while osteoclast-derived signals can influence osteoblast activity and bone formation [3]. When this balance is disrupted, it leads to various pathological conditions such as osteoporosis [4,5], rheumatoid arthritis [6], and other metabolic bone diseases [7]. Excessive osteoclast-mediated bone resorption contributes to bone loss and osteoporosis, whereas impaired osteoblast activity can compromise bone formation and skeletal integrity [8].

The bone remodeling process is primarily regulated by the RANK–RANKL–OPG axis, which consists of RANK, RANKL, and OPG [9,10]. Within this axis, RANKL binds to its receptor RANK on osteoclast precursors to promote osteoclast differentiation and activation, while OPG acts as a decoy receptor that neutralizes RANKL and inhibits bone resorption. In contrast, the Wnt/β-catenin pathway serves as the principal signaling cascade controlling osteoblast differentiation and bone formation. Its activity is tightly regulated by inhibitors such as sclerostin to maintain skeletal homeostasis [11]. Besides these pathways, bone remodeling is further modulated by systemic hormonal regulation, inflammatory mediators, and mechanical loading [12].

Specifically, oxidative stress and chronic inflammation are now recognized as major factors disrupting the balance between bone formation and resorption. In bone cells, excessive generation of reactive oxygen species (ROS) promotes cellular degeneration and apoptosis. In addition, ROS activates nuclear factor-κB (NF-κB) signaling and upregulates RANKL expression, thereby stimulating osteoclast differentiation. In contrast, ROS suppresses osteoblast growth and function, leading to bone loss [13,14]. Thus, the regulation of ROS levels and inflammation is a crucial strategy in the management of bone diseases.

Among the endogenous defense systems that counteract oxidative stress, heme oxygenase-1 (HO-1) has attracted considerable attention because of its dual antioxidant and anti-inflammatory properties [15,16]. HO-1 generates carbon monoxide (CO), free ferrous iron (Fe^2+^), and biliverdin, which participate in cellular signaling, inflammatory regulation, and cell survival pathways [15,17]. Together, these bioactive heme breakdown products contribute to cellular redox regulation and homeostasis. Specifically, biliverdin is subsequently converted into the potent antioxidant bilirubin [18]. CO acts as a signaling molecule that attenuates inflammatory responses, and Fe^2+^ is sequestered by ferritin to prevent iron-related toxicity [18,19]. HO-1 expression is primarily regulated by the nuclear factor erythroid 2-related factor 2 (Nrf2) pathway. Under oxidative stress conditions, Nrf2 translocates to the nucleus and induces transcription of the HO-1 gene (*HMOX1*) [16].

**Figure 1:**
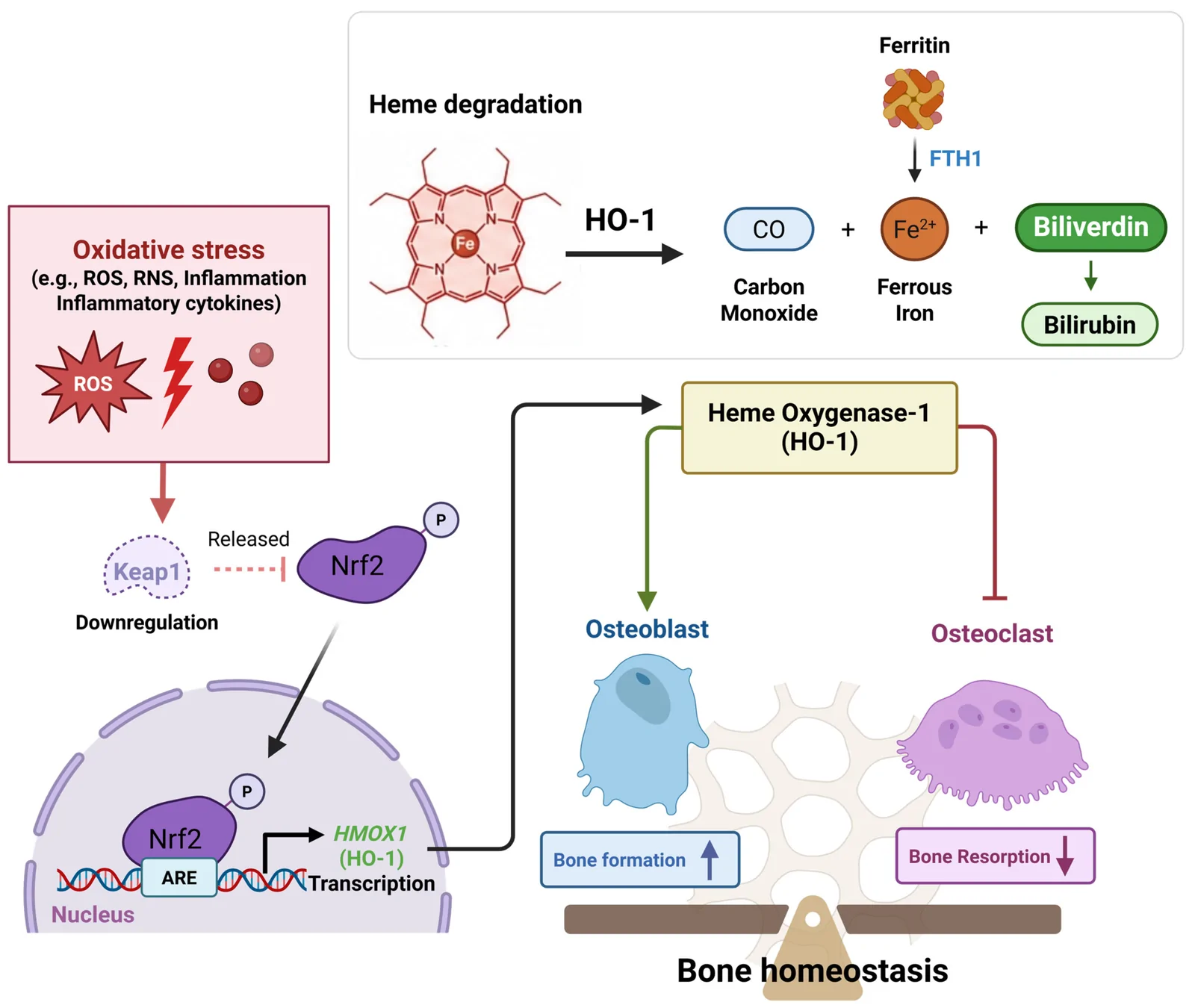
Overview of the role of heme oxygenase-1 (HO-1) in bone remodeling. Under oxidative stress and inflammation, Kelch-like ECH-associated protein 1 (Keap1) is inactivated, releasing nuclear factor erythroid 2-related factor 2 (Nrf2) for nuclear translocation where it binds antioxidant response elements (AREs) in the *HMOX1* promoter to drive HO-1 expression. HO-1 catalyzes heme degradation into three bioactive metabolites: carbon monoxide (CO), biliverdin subsequently reduced to bilirubin, and ferrous iron (Fe^2+^) sequestered by ferritin heavy chain (FTH1). Through generation of these products, HO-1 promotes osteoblast-mediated bone formation while restraining osteoclast-mediated bone resorption, collectively supporting bone homeostasis. Green arrows, activation/promotion; red lines, inhibition/suppression; red dashed line, inactivation; upward arrows, increased expression or activity; downward arrows, decreased expression or activity. Created in BioRender. Apichai, S. (2026) https://BioRender.com/oodlant (accessed on 20 August 2026).

Recent advances have substantially expanded our understanding of HO-1 beyond its classical antioxidant and anti-inflammatory functions. Emerging evidence indicates that HO-1 participates in multiple aspects of bone remodeling, including osteoblast differentiation, osteoclastogenesis, mitochondrial homeostasis, cellular senescence, iron metabolism, ferroptosis, and bone–immune interactions [20,21] (Figure 1). Despite growing interest in these areas, a comprehensive framework integrating the cell-specific and signaling functions of HO-1 in skeletal biology is still lacking. Therefore, this review is intended to provide a mechanistic overview of how HO-1 regulates bone remodeling, with focus on its modulation of cellular and molecular pathways involved in bone biology. The therapeutic potential of HO-1 in bone-related disorders is also discussed.

## 2. Molecular Actions of HO-1 in Bone Formation

### 2.1. Role of HO-1 in Osteogenesis

#### 2.1.1. Effects of HO-1 on Osteoblast Differentiation and Function

The transition from mesenchymal progenitor cells to mature, matrix-producing osteoblasts is a highly coordinated process regulated by multiple transcription factors mediating distinct redox-dependent signaling pathways [22]. Increasing evidence indicates that HO-1 plays an important regulatory role in osteoblast differentiation and function by modulating redox responses, transcriptional programs, and intracellular metabolic balance [20]. Induction of HO-1 enhances osteogenic differentiation and promotes the expression of osteogenic markers, including alkaline phosphatase (ALP), osteocalcin, and osteonectin, while favoring osteoblast lineage commitment over adipogenesis in mesenchymal stem cells [23,24].

HO-1 influences osteoblast differentiation primarily by regulating osteogenic transcription factors and related signaling cascades. HO-1 induction stimulates the expression of key osteogenic regulators such as runt-related transcription factor 2 (Runx2), distal-less homeobox 5 (Dlx5), and osteocalcin, which are essential for osteoblast maturation and bone matrix formation [25]. In addition, HO-1 expression is closely linked to the upregulation of bone morphogenetic protein-2 (BMP-2), which promotes the phosphorylation of Smad1/5/9 and activates downstream osteogenic gene transcription [26].

Angiogenesis and osteogenesis are tightly coupled processes during bone development and regeneration. Hypoxia-inducible factor 1-alpha (HIF-1α) is a principal regulator of vascular endothelial growth factor (VEGF) transcription under hypoxic conditions. In the HIF-1α-VEGF axis, HO-1 functions as an important downstream mediator. Thus, osteoblast-secreted VEGF promotes the proliferation and osteogenic differentiation of bone marrow stromal cells (BMSCs) by upregulating HO-1 expression, and pharmacological blockade of HO-1 using Tin protoporphyrin IX (SnPP) abolishes these VEGF-driven effects [27]. Of interest, HO-1 and VEGF cooperate reciprocally with each other. Thus, CO generated from HO-1-mediated heme degradation induces VEGF synthesis through p38 mitogen-activated protein kinase (MAPK)-dependent activation of the transcription factor Sp1. It also stabilizes HIF-1α protein, thereby sustaining proangiogenic gene expression [28]. Conversely, induction of HO-1 in endothelial cells increases VEGF production and enhances angiogenic activity, further supporting the involvement of HO-1 in angiogenic signaling [29].

#### 2.1.2. Cytoprotective Functions of HO-1 in Osteoblasts

HO-1 activation is typically accompanied by the induction of ferritin, particularly ferritin heavy chain (FTH1), which sequesters free ferrous iron (Fe^2+^) generated during heme degradation and prevents iron-mediated oxidative damage through the Fenton reaction [20]. This coordinated response helps maintain intracellular redox balance during osteoblast differentiation [30]. While iron overload has been shown to inhibit osteogenic commitment of mesenchymal stem cells through ferritin-mediated suppression of Runx2 expression [30], HO-1 induction counteracts this by limiting free iron availability and attenuating oxidative stress [31].

Osteoblasts possess high mitochondrial density to meet the ATP demands of protein synthesis and calcium transport. Mitochondrial dynamics, governed by the balance between fusion (mediated by Mfn1/2 and Opa1) and fission (mediated by Drp1), are critical for the osteoblast function; excessive Drp1-dependent fission under oxidative stress has been shown to impair osteoblast differentiation and mineralization [32]. By reducing intracellular ROS levels, HO-1 may preserve this fusion–fission balance and maintain mitochondrial integrity under pathological conditions [33]. Furthermore, mitochondrial biogenesis in osteoblasts is regulated by the SIRT3/PGC-1α/TFAM axis, which is essential for maintaining bioenergetic capacity during differentiation [34].

**Figure 2:**
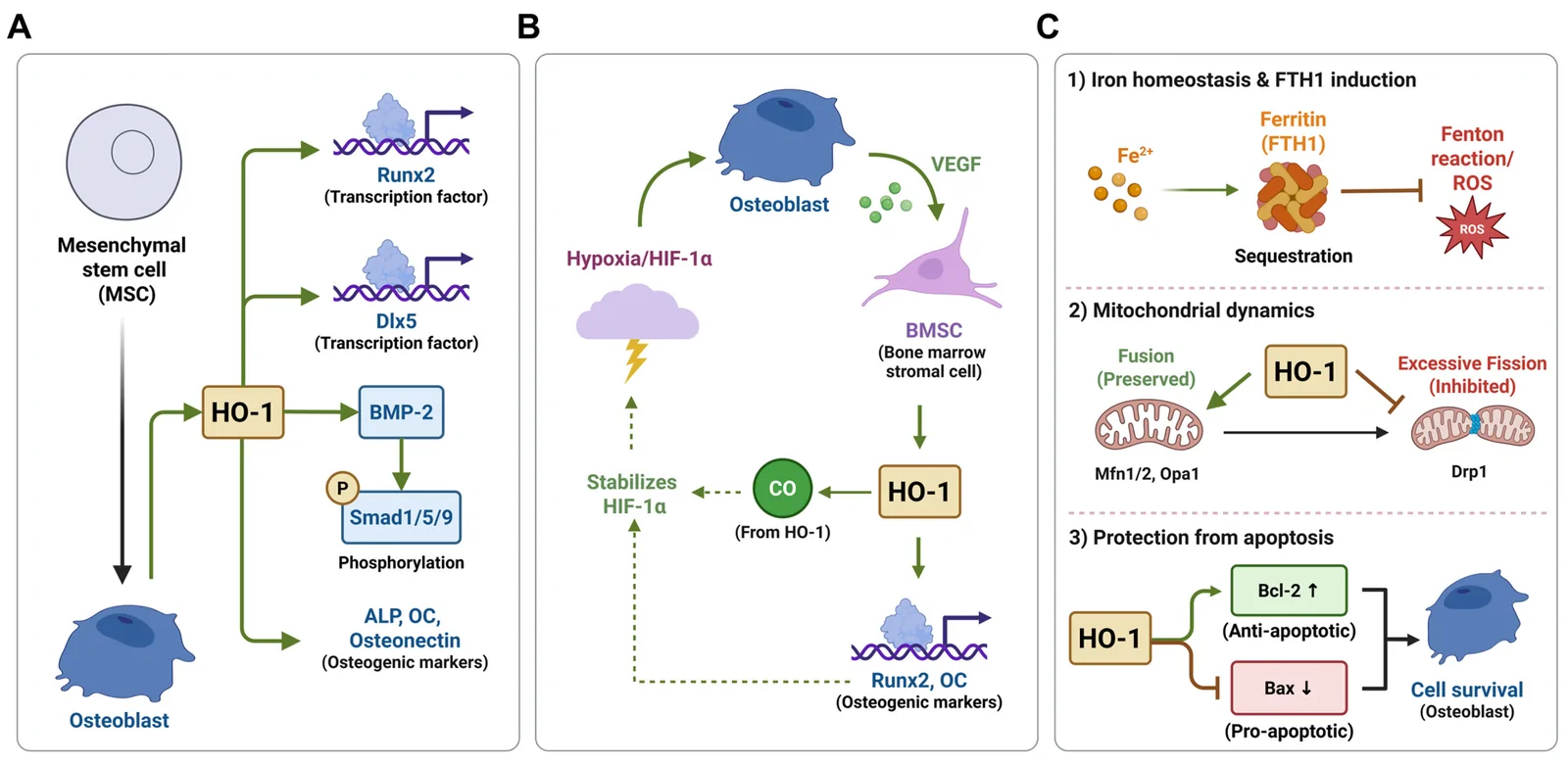
Molecular mechanisms of HO-1 in osteoblast differentiation and cytoprotection. (**A**) HO-1 promotes osteoblast differentiation from mesenchymal stem cells (MSCs) by upregulating osteogenic transcription factors Runx2 and Dlx5, enhancing BMP-2-mediated phosphorylation of Smad1/5/9, and increasing expression of osteogenic markers alkaline phosphatase (ALP), osteocalcin (OC), and osteonectin. (**B**) Within the angiogenic–osteogenic crosstalk axis, hypoxia-inducible factor-1α (HIF-1α) drives osteoblast-derived vascular endothelial growth factor (VEGF) secretion, which upregulates HO-1 in bone marrow stromal cells (BMSCs) and promotes osteogenic differentiation via Runx2 and OC; HO-1-derived carbon monoxide (CO) and Runx2 may in turn stabilize HIF-1α, sustaining VEGF expression through a positive feedback loop. (**C**) HO-1 exerts cytoprotective effects through three mechanisms: (1) induction of ferritin heavy chain (FTH1) to sequester ferrous iron (Fe^2+^) and prevent Fenton reaction-mediated reactive oxygen species (ROS) generation; (2) preservation of mitochondrial fusion (Mfn1/2, Opa1) over excessive fission (Drp1); and (3) suppression of pro-apoptotic Bax while promoting anti-apoptotic Bcl-2, thereby protecting osteoblasts from apoptosis. Green arrows, activation/promotion; red lines, inhibition/suppression; dashed arrows, indirect or proposed mechanisms; upward arrows, increased expression or activity; downward arrows, decreased expression or activity. Created in BioRender. Apichai, S. (2026) https://BioRender.com/l1pqbys (accessed on 20 August 2026).

Glucocorticoid exposure is known to induce osteoblast apoptosis by up-regulating the pro-apoptotic protein Bim [35]. However, HO-1 induction effectively protects osteoblasts from glucocorticoid-induced apoptotic death by suppressing Bax while promoting Bcl-2 [36]. By preserving osteoblast viability under pathological conditions, HO-1 has therapeutic potential in osteoporosis [37]. Figure 2 illustrates the role of HO-1 in differentiation of osteoblasts and their viability.

#### 2.1.3. HO-1 in Osteocyte Biology

Osteocytes are the most abundant cells in mature bone. They serve as key regulators of bone remodeling by coordinating signals between osteoblasts and osteoclasts [38]. Through regulation of OPG and RANKL, osteocytes influence osteoclastogenesis [39], while secretion of sclerostin modulates Wnt/β-catenin signaling and osteoblast activity [38,39]. Osteocytes also function as mechanosensors, translating mechanical stimuli into molecular signals that regulate bone adaptation and remodeling [40]. Emerging evidence suggests that HO-1 may participate in osteocyte responses to metabolic and oxidative stress. In particular, dysregulated HO-1 signaling has been implicated in osteocyte ferroptosis under diabetic conditions, linking HO-1 to osteocyte survival and pathological bone loss [41]. However, direct evidence supporting the roles of HO-1 in osteocyte-mediated RANKL regulation, sclerostin signaling, and mechanotransduction remains limited, warranting further investigation.

### 2.2. Regulatory Signaling Networks Governing HO-1 Activity in Bone Formation

#### 2.2.1. Nrf2/Bach1-Mediated Regulation of HO-1

Under basal conditions, Bach1 binds to antioxidant response elements (AREs) within the *HMOX1* promoter and suppresses HO-1 transcription. When cells are challenged with oxidative stress, Bach1 dissociates from the promoter, permitting Nrf2 to bind ARE sequences and induce HO-1 expression [42]. In osteoblasts, Bach1 silencing enhanced Nrf2/ARE signaling, upregulated HO-1 expression, and attenuated oxidative stress-induced apoptosis and dysfunction, highlighting the critical role of the Bach1/Nrf2/ARE axis in bone homeostasis [43]. Activation of the Nrf2/HO-1 pathway thus represents a major adaptive response to oxidative stress in bone cells.

The activity of Nrf2 is further regulated by post-translational modifications that influence its stability, nuclear localization, and transcriptional activity. Several MAPK family members, including ERK1/2 and p38 MAPK, have been implicated in the regulation of Nrf2 activation under oxidative stress conditions, facilitating its nuclear translocation and transcriptional activity [44,45]. In contrast, GSK-3β phosphorylates Nrf2 at the Neh6 domain, creating a phosphodegron recognized by the β-TrCP E3 ubiquitin ligase complex and targeting Nrf2 for proteasomal degradation [46]. GSK-3β therefore acts as a negative regulator of Nrf2, whereas inhibition of GSK-3β stabilizes Nrf2 and enhances the expression of downstream antioxidant genes, including HO-1. In addition, cobalt protoporphyrin IX (CoPP) has been reported to promote Bach1 degradation while stabilizing Nrf2 protein, leading to HO-1 upregulation through post-transcriptional regulatory mechanisms [47].

#### 2.2.2. Regulation by Long Non-Coding RNAs (lncRNAs)

Emerging evidence indicates that long non-coding RNAs (lncRNAs) function as important epigenetic regulators of oxidative stress-responsive signaling pathways, including the Nrf2/HO-1 axis [48,49]. In osteoblastic cells, dexamethasone has been reported to suppress CRNDE expression, resulting in increased oxidative stress and impaired osteogenic differentiation [24]. Restoration of CRNDE expression activated the Nrf2/HO-1 pathway and enhanced the expression of antioxidant enzymes and osteogenic markers such as Runx2, osteocalcin, and collagen type I [24]. Collectively, lncRNA-mediated regulation of the Nrf2/HO-1 pathway represents an additional epigenetic mechanism governing osteoblast function and redox homeostasis.

#### 2.2.3. Crosstalk Between HO-1 and Wnt/β-Catenin Signaling

The Wnt/β-catenin pathway is a central regulator of osteoblast differentiation and bone formation. Under conditions of oxidative stress, activated FOXO transcription factors can divert β-catenin from TCF/LEF-mediated transcription toward FOXO-dependent gene expression, thereby prioritizing stress resistance at the expense of osteogenic signaling [50,51]. In osteoprogenitor cells, this shift has been associated with reduced Cyclin D1 expression, impaired cell proliferation, and decreased bone formation, whereas genetic deletion of FOXOs enhances Wnt/β-catenin activity and preserves bone mass during aging [52]. Given the antioxidant functions of HO-1, reduction of intracellular ROS accumulation by HO-1 may help preserve β-catenin availability for canonical Wnt signaling, thereby supporting osteogenic activity.

GSK-3β functions as a common negative regulator of both the Wnt/β-catenin and Nrf2 signaling pathways. Activation of GSK-3β promotes β-catenin degradation and facilitates β-TrCP-mediated degradation of Nrf2, leading to repression of osteogenic and antioxidant responses [46,53]. Conversely, inhibition of GSK-3β stabilizes both β-catenin and Nrf2, supporting the coordinated activation of osteogenic differentiation and cellular defense mechanisms. This convergence suggests a potential mechanistic link through which HO-1-associated antioxidant signaling may indirectly influence osteogenic pathways.

**Figure 3:**
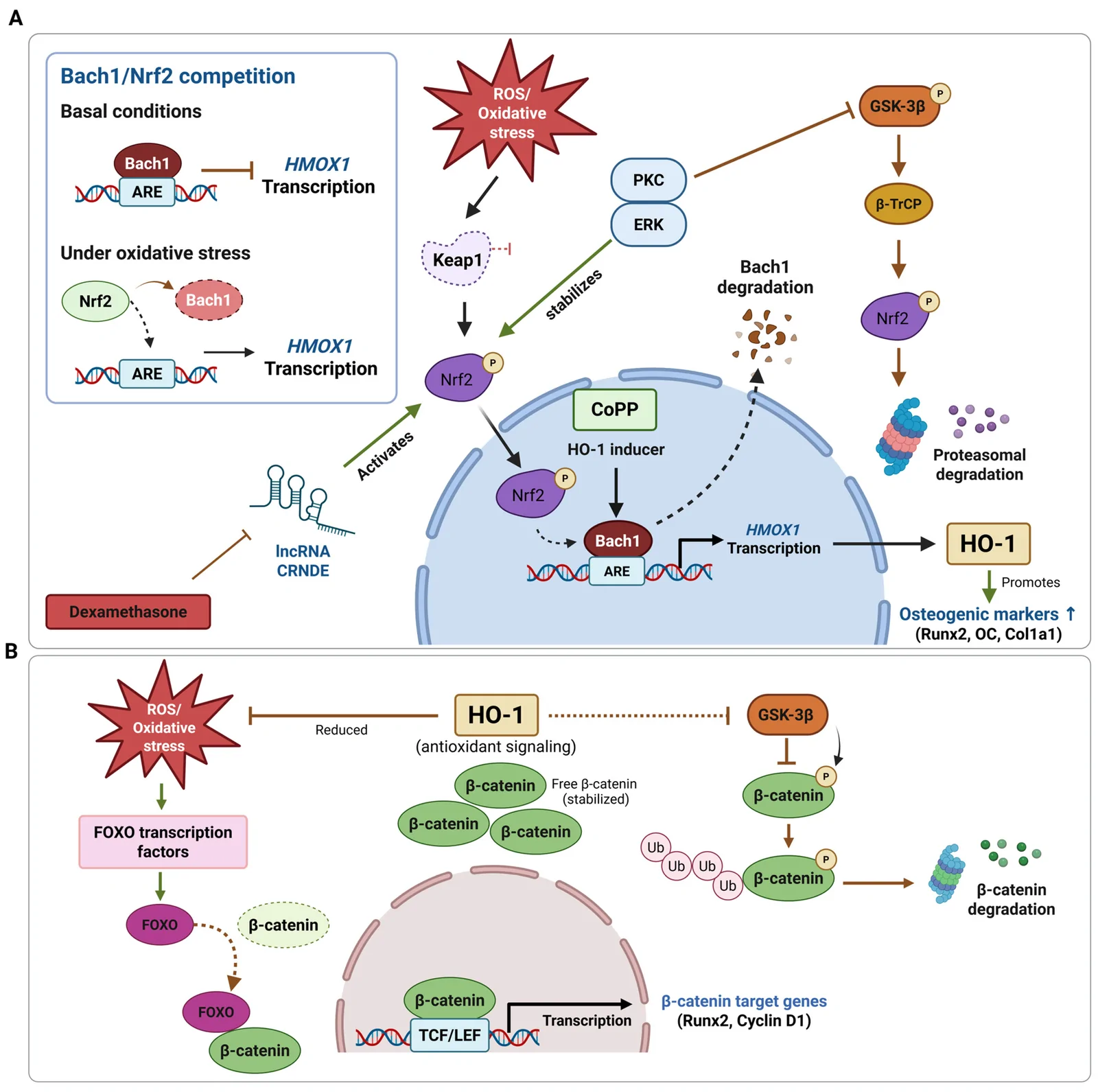
Regulatory signaling networks associated with HO-1 during osteogenesis. (**A**) *HMOX1* expression is regulated through multiple mechanisms, including Nrf2/Bach1 competition, PKC/ERK-mediated Nrf2 stabilization, GSK-3β/β-TrCP-dependent Nrf2 degradation, and cobalt protoporphyrin IX (CoPP)-induced Bach1 degradation. An inset illustrates epigenetic regulation by the long non-coding RNA (lncRNA) CRNDE, which is suppressed by dexamethasone and positively regulates the Nrf2/HO-1 pathway, resulting in increased expression of osteogenic markers, including Runx2, osteocalcin (OC), and collagen type I alpha 1 (Col1a1). (**B**) HO-1-associated antioxidant signaling intersects with canonical Wnt/β-catenin signaling through two complementary mechanisms. By reducing oxidative stress, HO-1 may preserve β-catenin availability for TCF/LEF-dependent transcription by limiting FOXO-mediated competition. In addition, HO-1-associated antioxidant signaling may indirectly inhibit GSK-3β, thereby stabilizing β-catenin and supporting expression of β-catenin target genes, including Runx2 and Cyclin D1. Green arrows, activation/promotion; red lines, inhibition/suppression; dashed arrows, indirect or proposed mechanisms; upward arrows, increased expression or activity; downward arrows, decreased expression or activity. Created in BioRender. Apichai, S. (2026) https://BioRender.com/11xs6kf (accessed on 20 August 2026).

The representative intracellular signaling pathways responsible for HO-1-mediated osteogenesis is shown in Figure 3.

## 3. HO-1 and Bone Resorption

### 3.1. Regulation of Osteoclastogenesis by HO-1

#### 3.1.1. Effects of HO-1 on Osteoclast Differentiation and Function

HO-1 exerts predominantly inhibitory effects on osteoclastogenesis and bone-resorptive activity. Induction of HO-1 by hemin suppresses osteoclast formation in a dose-dependent manner, with osteoclast precursors appearing more susceptible than mature osteoclasts to HO-1-mediated regulation [54]. HO-1 deficiency is accompanied by the increased number of osteoclasts and their activity together with reduced bone mass, corroborating a role for endogenous HO-1 in limiting excessive bone resorption [55].

RANKL may further amplify osteoclastogenesis by suppressing HO-1 expression in osteoclast precursors, thereby promoting high mobility group box 1 (HMGB1) release; restoration of HO-1 expression through pharmacological induction attenuates this effect and inhibits osteoclast formation [56]. HO-1 expression is additionally subject to transcriptional repression by Krüppel-like factor 7 (KLF7), and suppression of HO-1 through KLF7 overexpression enhances osteoclast differentiation in a manner reversible by hemin-mediated HO-1 induction [57].

The contribution of HO-1 to osteoclastogenesis may vary according to the differentiation stage of osteoclast lineage cells. In bone marrow macrophages, loss of HO-1 through genetic deletion or siRNA-mediated silencing reduced RANKL-induced tartrate-resistant acid phosphatase (TRAP)-positive cell formation and attenuated NFATc1 and cathepsin K expression, whereas HO-1 knockdown after RANKL priming exerted minimal effects on osteoclast marker expression [58]. Despite these stage-dependent observations, elevated circulating TRAP5b levels in HO-1-deficient mice support an overall inhibitory role of endogenous HO-1 in osteoclast formation [58].

#### 3.1.2. Regulation of RANKL/RANK/NFATc1 Signaling

HO-1 interferes with multiple components of the RANKL signaling cascade that governs osteoclast differentiation. Induction of HO-1 suppresses the expression of c-Fms, RANK, TRAF6, and cFos, whereas HO-1 deficiency enhances NF-κB and NFATc1 activation following RANKL stimulation [54,55]. HO-1 deficiency is also accompanied by increased intracellular calcium accumulation in osteoclast precursors, an effect that may potentiate NFATc1 autoamplification, further supporting a role for HO-1 in limiting signal propagation during osteoclastogenesis [55].

Upregulation of HO-1 by deltamethrin reduced NFATc1 protein levels and concomitantly suppressed the expression of Src and cathepsin K in bone marrow-derived macrophages and RAW-D cells [59]. Nrf2-dependent induction of HO-1 by magnolol also suppresses NFATc1 nuclear translocation and MMP-9 activity in osteoclast precursors, and pharmacological inhibition of HO-1 with tin protoporphyrin IX (SnPP) largely abolishes these anti-osteoclastogenic effects [60]. HO-1-derived CO additionally reduces NFATc1 expression through inhibition of IKK-dependent NF-κB activation, and thereby attenuates NFATc1-driven osteoclastogenesis [61].

#### 3.1.3. ROS and Redox Control During Osteoclastogenesis

ROS function as intracellular signaling mediators during osteoclastogenesis, and excessive ROS accumulation amplifies osteoclastogenic signaling. As an inducible antioxidant enzyme, HO-1 counteracts this process by preserving intracellular redox homeostasis. HO-1 deficiency is associated with elevated systemic and intracellular ROS levels accompanied by augmentation of NF-κB and NFATc1 activation and increased osteoclast formation [55].

In osteoarthritic joints, downregulation of *HMOX1* expression emerges as a characteristic feature of activated osteoclasts in subchondral bone, pointing to a pathophysiological role for HO-1 in limiting osteoclast-mediated bone resorption [62]. Activation of the Nrf2/HO-1 axis by salvianolic acid A (SAA) has also been linked to suppression of MAPK-dependent osteoclastogenic signaling; SAA attenuated ROS accumulation and reduced NF-κB and MAPK activation, resulting in decreased NFATc1 expression and impaired osteoclast differentiation both in cultured cells and in ovariectomized mice [63].

HO-1-derived by-products further contribute to the regulation of redox-sensitive osteoclastogenic signaling. CO suppresses RANKL-induced osteoclastogenesis and osteoclastic resorptive activity by inhibiting ROS production and IKK-dependent NF-κB activation, thereby reducing NFATc1 expression, whereas bilirubin exerts weaker inhibitory effects on osteoclast differentiation [61].

**Figure 4:**
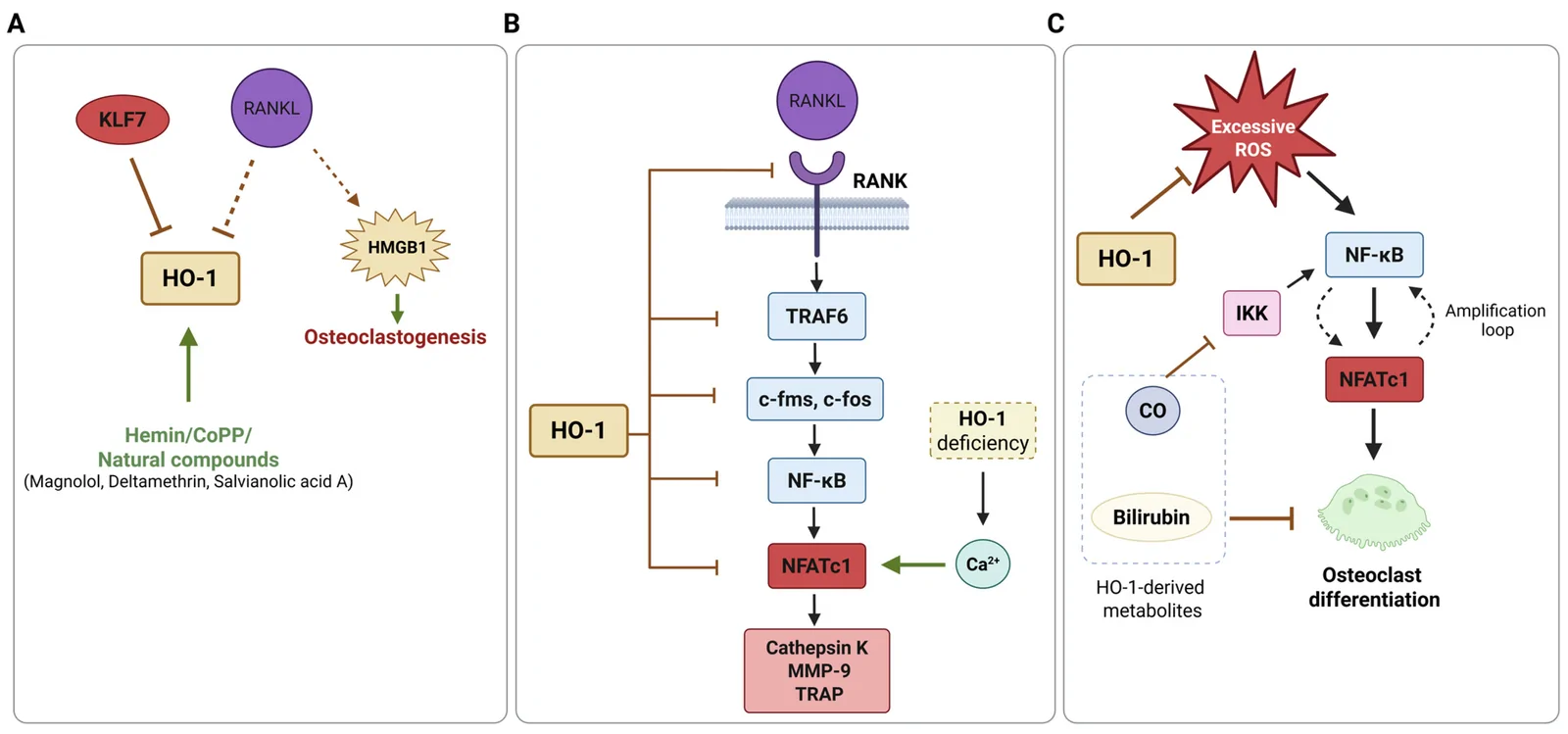
Molecular mechanisms by which HO-1 suppresses osteoclastogenesis and bone resorption. (**A**) HO-1 expression is negatively regulated by Krüppel-like factor 7 (KLF7) and by RANKL-mediated suppression; hemin, cobalt protoporphyrin IX (CoPP), and selected natural compounds (magnolol, deltamethrin, and salvianolic acid A) restore HO-1 expression. RANKL-mediated suppression of HO-1 promotes high mobility group box 1 (HMGB1) release and amplifies osteoclastogenesis. (**B**) HO-1 suppresses RANKL-induced osteoclastogenic signaling by inhibiting multiple components of the RANKL/RANK/NF-κB/NFATc1 axis, including TRAF6, c-fms, c-fos, nuclear factor-κB (NF-κB), and nuclear factor of activated T cells 1 (NFATc1), resulting in reduced expression of cathepsin K, matrix metalloproteinase-9 (MMP-9), and tartrate-resistant acid phosphatase (TRAP). HO-1 deficiency may also increase intracellular Ca^2+^ accumulation, further enhancing NFATc1 autoamplification. (**C**) By preserving intracellular redox homeostasis, HO-1 attenuates ROS-driven NF-κB/NFATc1 activation. HO-1-derived carbon monoxide (CO) inhibits inhibitor of κB kinase (IKK)-dependent NF-κB activation, whereas bilirubin suppresses osteoclast differentiation. Green arrows, activation/promotion; red/brown lines, inhibition/suppression; dashed arrows, indirect or proposed mechanisms; upward arrows, increased expression or activity; downward arrows, decreased expression or activity. Created in BioRender. Apichai, S. (2026) https://BioRender.com/5hmyjzx (accessed on 20 August 2026).

The inhibitory effects of HO-1 on osteoclastogenesis and bone-resorptive activity are depicted in Figure 4.

### 3.2. Anti-Resorptive Mechanisms of HO-1

#### 3.2.1. Modulation of Inflammatory Signaling

Inflammatory cytokines, particularly TNF-α, IL-1β, and IL-6, promote bone resorption through multiple mechanisms. TNF-α and IL-1β enhance RANKL expression in osteoblasts and stromal cells and can directly stimulate osteoclast precursor differentiation under permissive osteoclastogenic conditions [64,65]. IL-6 can promote osteoclastogenesis, although its effects on osteoclast precursors may depend on the local level of RANKL and the mode of IL-6 signaling [66,67].

HO-1 exerts broad anti-inflammatory effects that counteract the cytokine-driven resorptive processes. Through inhibition of NF-κB nuclear translocation and attenuation of upstream MAPK signaling, HO-1 induction reduced production of TNF-α, IL-1β, and IL-6, potentially limiting the pro-resorptive cytokine milieu that supports osteoclastogenesis [20,37]. Pharmacological induction of HO-1 ameliorated both joint inflammation and periarticular bone erosion in inflammatory arthritis models. This suggests that HO-1-mediated suppression of inflammatory signaling may contribute to protection of skeletal integrity under pathological conditions [54]. Activation of the Nrf2/HO-1 axis has been associated with reduced circulating levels of TNF-α, IL-1β, and IL-6 together with increased serum OPG levels in ovariectomized mice, further corroborating a role for HO-1 in restraining inflammation-driven bone loss [68].

#### 3.2.2. Crosstalk Between Osteoblasts and Osteoclasts

Bone remodeling occurs within the basic multicellular unit (BMU) in which osteoclast-mediated resorption is tightly coupled to subsequent osteoblast-mediated bone formation [69,70]. Disruption of this coupling contributes to pathological bone loss, whereas preservation of coordinated signaling between osteoblasts and osteoclasts is essential for skeletal homeostasis. Through its concurrent involvement in both cell populations, HO-1 may contribute to the maintenance of balanced bone remodeling [20,37]. However, direct evidence linking HO-1 to coupling mechanisms within the BMU remains limited. At the molecular level, HO-1-derived CO has been shown to suppress RANKL-induced NFATc1 activation in osteoclast precursors in vitro [61]. Conversely, RANKL has been reported to induce suppression of HO-1 in osteoclast precursors [56]. It is also possible that osteoblast-derived signals capable of sustaining HO-1 expression may indirectly attenuate osteoclastogenic responsiveness, adding a potential paracrine dimension to osteoblast–osteoclast crosstalk in the context of HO-1 biology.

**Figure 5:**
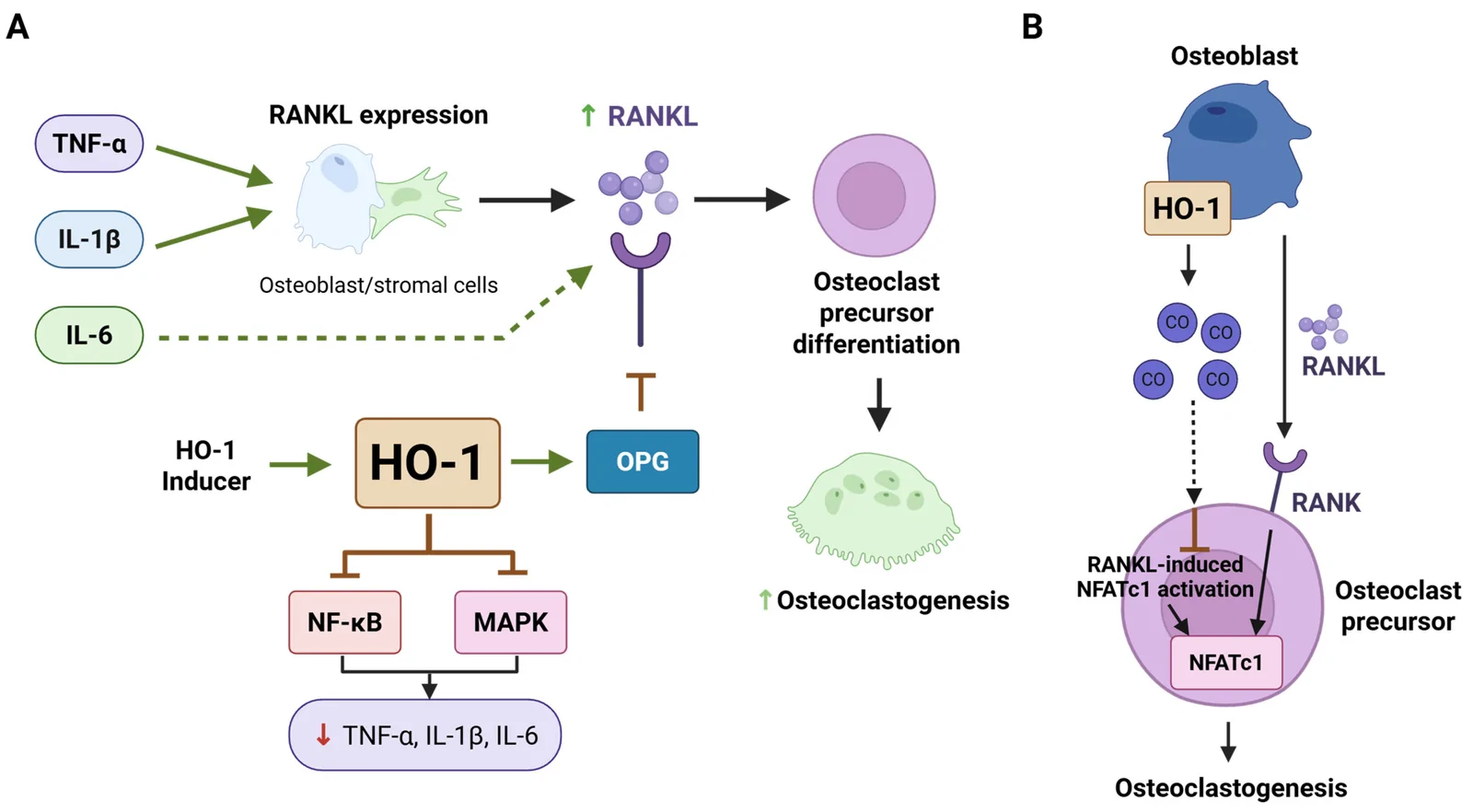
Anti-resorptive mechanisms of HO-1 through modulation of inflammatory signaling and osteoblast–osteoclast crosstalk. (**A**) Pro-inflammatory cytokines, including tumor necrosis factor-α (TNF-α), interleukin-1β (IL-1β), and interleukin-6 (IL-6), enhance receptor activator of nuclear factor-κB ligand (RANKL) expression and promote osteoclastogenesis. HO-1 suppresses nuclear factor-κB (NF-κB) and mitogen-activated protein kinase (MAPK) signaling, reducing TNF-α, IL-1β, and IL-6 production while increasing osteoprotegerin (OPG) levels to limit RANKL-mediated osteoclast differentiation. (**B**) Within the basic multicellular unit (BMU), osteoblast-derived carbon monoxide (CO) may act as a paracrine signal that suppresses RANKL-induced nuclear factor of activated T cells 1 (NFATc1) activation in osteoclast precursors. RANKL also suppresses HO-1 expression in osteoclast precursors, supporting an additional regulatory interaction between osteoblasts and osteoclasts during bone remodeling. Green arrows, activation/promotion; red/brown lines, inhibition/suppression; black arrows, signal transduction or molecular flow; dashed arrows, indirect or proposed mechanisms; upward arrows, increased expression or activity; downward arrows, decreased expression or activity. Created in BioRender. Apichai, S. (2026) https://BioRender.com/04lxf0j (accessed on 20 August 2026).

The involvement of HO-1 in repression of bone resorption and underlying mechanisms with focus on modulation of inflammatory signaling and osteoblast–osteoclast crosstalk is schematically represented in Figure 5.

## 4. Therapeutic Implications of HO-1 Modulation in Bone Disease

### 4.1. Pathological Contexts of HO-1 Dysregulation in Bone Loss

HO-1, a stress-responsive cytoprotective enzyme induced by oxidative and pro-inflammatory stimuli, plays a central role in maintaining redox homeostasis during bone remodeling. Under pathological conditions such as estrogen deficiency, aging, iron overload, diabetes, glucocorticoid exposure, and chronic inflammatory disorders, excessive oxidative stress and persistent inflammation disrupt HO-1 regulation, which provokes impaired bone remodeling and skeletal deterioration.

In postmenopausal osteoporosis, estrogen withdrawal amplifies oxidative stress and NF-κB-driven RANKL expression; this suppresses HO-1 induction and consequently dampens endogenous antioxidant defenses that normally limit osteoclast differentiation [20,37]. Glucocorticoid-induced osteoporosis represents a distinct pathological context in which HO-1 suppression is well documented. Glucocorticoids suppress HO-1 expression and promote osteoblast apoptosis by disrupting the balance between pro- and anti-apoptotic signaling, whereas pharmacological induction of HO-1 expression preserves osteoblast viability through ERK1/2-dependent signaling [36]. Inflammatory bone diseases, including rheumatoid arthritis [71] and lipopolysaccharide (LPS)-induced inflammatory bone loss [54], represent other pathological contexts in which HO-1 plays a protective role. HO-1 induction suppresses inflammatory signaling mediated by ROS-dependent NF-κB activation, which inhibits osteoclastogenesis, and limits inflammatory bone destruction [54,71].

However, HO-1 exerts an opposite effect in murine models of diabetic osteoporosis. Thus, Nrf2/c-Jun-dependent transcriptional activation of *HMOX1* in osteocytes from experimentally induced diabetic mice contributes to iron dysregulation and ferroptotic cell death [41]. Pharmacological inhibition of HO-1 or ferroptosis alleviates trabecular bone deterioration in experimental diabetic osteoporosis, highlighting the dual role of HO-1, depending on disease context [41].

### 4.2. Pharmacological Strategies Targeting HO-1 in Bone Disease

Pharmacological modulation of HO-1 has attracted considerable interest as a strategy to counteract oxidative stress-associated bone loss. Current approaches can be broadly categorized into direct HO-1 inducers, natural compounds, existing drugs with HO-1-modulating activity, and emerging HO-1-targeting therapeutics (Table 1 and Table 2). Although these agents act through distinct mechanisms, most ultimately enhance/potentiate HO-1 signaling or its downstream cytoprotective effects, thereby improving osteoblast function, suppressing osteoclast differentiation, or both.

#### 4.2.1. Direct HO-1 Inducers

Direct pharmacological induction of HO-1 has been instrumental in defining the biological functions of this cytoprotective enzyme in bone remodeling. Hemin suppresses osteoclast differentiation and inflammatory bone resorption, whereas CoPP protects osteoblasts against glucocorticoid-induced oxidative injury through HO-1-dependent mechanisms [36,54]. The effects of both HO-1 inducers on bone remodeling and homeostasis and underlying mechanisms are summarized in Table 1.

#### 4.2.2. Natural Compounds

Natural compounds constitute the largest category of HO-1-modulating agents investigated in bone research. Despite their structural diversity, most enhance HO-1-associated antioxidant signaling through activation of Nrf2, thereby promoting osteoblast differentiation, preserving osteoblast viability, and suppressing osteoclastogenesis. As listed in Table 1, curcumin, gomisin A, and neobavaisoflavone have been shown to protect osteogenic cells from oxidative stress or glucocorticoid-induced injury while stimulating osteogenic differentiation through HO-1-dependent mechanisms [24,72,73].

In addition to their osteogenic effects, several natural compounds predominantly inhibit osteoclast differentiation and bone resorption. Magnolol, puerarin, schisandrin A, salvianolic acid A, and theaflavin-3,3′-digallate suppress RANKL-mediated osteoclastogenesis by attenuating ROS-dependent signaling pathways involving NF-κB and NFATc1, thereby limiting bone resorption in cellular and experimental models [60,63,74,75,76]. More recently, picein has been reported to promote bone regeneration by simultaneously inhibiting ferroptosis and enhancing osteogenic differentiation, and this has been attributed to activation of the Nrf2/HO-1/GPX4 axis [77].

**Table 1:** Effects of some modulators and products of HO-1 on bone remodeling.

Agent	HO-1-Related Mechanism	Experimental Model	Biological Effects	[Ref.]
Direct HO-1 inducers				
Hemin	Induction of HO-1 through MAPK activation, leading to suppression of c-Fms/RANK/TRAF6/c-Fos signaling	Murine spleen cells CD11b^+^ osteoclast precursors LPS-induced calvarial bone loss mice TNF-transgenic arthritis mice	↓ Osteoclast differentiation ↓ Osteoclast differentiation, ↓ Bone resorption ↓ Osteoclast number ↓ Inflammatory bone loss, ↓ Bone erosion	[54]
	HO-1 protein induction and carbon monoxide-mediated suppression of ROS-dependent NF-κB/NFATc1 signaling	RAW264.7 cells BMMs HO-1+/− mice	↓ Osteoclast differentiation ↓ Osteoclast differentiation, ↓ Bone resorption ↓ Osteoclast formation, ↓ Bone resorption	[61]
CoPP	Induction of HO-1 with activation of ERK1/2 signaling	Dex-treated MC3T3-E1	↓ ROS, ↓ Osteoblast apoptosis, ↑ Cell viability	[36]
Natural compounds				
Arecoline	HO-1-mediated inhibition of ferroptosis	MC3T3-E1 cells Zebrafish iron overload model	↓ Ferroptosis, ↑ Osteoblast differentiation ↑ Skeletal mineralization, ↓ Bone impairment	[31]
Chlorogenic acid	p21-mediated Nrf2/HO-1 activation	MC3T3-E1 cells	↓ Osteoblast apoptosis	[78]
Corynoline	Nrf2/HO-1 activation	Osteoblasts Dex-induced osteoporosis rats	↓ Oxidative stress, ↑ Osteogenic differentiation ↓ Bone loss	[79]
Costunolide	ATF4-mediated HO-1 activation	C3H10T1/2 cells	↑ Osteogenic differentiation, ↑ Matrix mineralization	[25]
Curcumin	Activation of HO-1 signaling pathway	Rat MSCs	↑ Osteogenic differentiation, ↓ Adipogenic differentiation	[72]
	Activation of Nrf2/HO-1-mediated antioxidant pathway	H_2_O_2_-treated mouse ASCs	↓ Apoptosis	[80]
Erxian decoction	Akt-mediated Nrf2/HO-1 activation	TNF-α-treated MC3T3-E1 cells OVX rats	↓ Osteoblast apoptosis ↓ Bone loss, ↑ BMD	[81]
Geniposide	Activation of Nrf2/HO-1-mediated antioxidant pathway	Cadmium-treated MC3T3-E1 cells	↓ Osteoblast apoptosis, ↑ Osteogenic differentiation	[82]
Glabridin	Activation of Nrf2/HO-1 signaling	Methylglyoxal-treated MC3T3-E1 cells	↓ Oxidative stress, ↑ Cell viability	[83]
Gomisin A	HO-1 induction-mediated maintenance of mitochondrial homeostasis	High glucose-treated MC3T3-E1 cells	↓ Oxidative stress, ↑ Osteogenic differentiation, ↑ Matrix mineralization	[73]
Lutein	Activation of Nrf2-dependent HO-1 and NQO1 expression with suppression of NF-κB/NFATc1 signaling	OVX rats	↓ Oxidative stress, ↓ Inflammatory responses, ↓ Osteoclast-specific marker expression	[84]
Magnolol	HO-1-dependent inhibition of NFATc1 via Nrf2 activation	RAW264.7 cells	↓ Osteoclast differentiation, ↓ Bone resorption	[60]
Neobavaisoflavone	CRNDE-mediated activation of Nrf2/HO-1 signaling	Dex-treated MC3T3-E1 cells	↓ Osteoblast apoptosis, ↓ Oxidative stress, ↑ Osteogenic differentiation	[24]
Picein	Activation of Nrf2/HO-1/GPX4 signaling	Erastin-treated rat BMSCs OVX rat femoral bone defect model	↑ Osteogenic differentiation, ↓ Ferroptosis ↑ Bone regeneration	[77]
Pristimerin	Activation of Nrf2/HO-1 signaling	Mouse BMMs OVX mice	↓ Osteoclast differentiation, ↓ Bone resorption ↓ Bone loss, ↓ Osteoclast number	[68]
Puerarin	HO-1-mediated inhibition of TRAF6/ROS-dependent MAPK/NF-κB signaling	Mouse BMMs RAW264.7 cells OVX mice	↓ Osteoclast differentiation, ↓ Bone resorption ↓ ROS, ↓ Osteoclast differentiation, ↓ Bone resorption ↑ BMD, ↓ Bone loss, ↓ Osteoclast number	[74]
Quercetin	Modulation of HO-1-mediated oxidative stress response	Fetal rat calvarial osteoblasts	↓ Oxidative stress, ↑ Osteogenic differentiation, ↑ Matrix mineralization	[85]
Resveratrol	Nrf2-mediated HO-1 induction	LPS-stimulated human gingival fibroblasts Ligature/LPS-induced periodontitis rats	↓ Inflammatory responses, ↓ Oxidative stress ↓ Alveolar bone loss, ↓ Osteoclast formation	[86]
Salvianolic acid A	Activation of Nrf2/HO-1 signaling with suppression of ROS-dependent osteoclastogenesis	Primary BMMs RAW264.7 cells OVX mice	↓ Osteoclast differentiation, ↓ Bone resorption ↓ ROS, ↓ Osteoclast differentiation, ↓ Bone resorption ↓ Bone loss, ↓ Osteoclast number	[63]
Schisandrin A	Activation of Nrf2/HO-1 signaling	Mouse BMMs OVX mice	↓ Osteoclast differentiation, ↓ Bone resorption ↓ Bone loss	[75]
Theaflavin-3,3′-digallate	Activation of Nrf2/HO-1 signaling with suppression of MAPK phosphorylation and ROS-dependent osteoclastogenesis	Primary BMMs OVX mice	↓ Osteoclast differentiation, ↓ F-actin ring formation ↓ Bone loss, ↓ Osteoclast number	[76]
2,3,5,4′-Tetrahydroxystilbene-2-O-β-D-glycoside	Activation of Nrf2/HO-1 signaling with suppression of NF-κB signaling	H_2_O_2_-treated MC3T3-E1 pre-osteoblasts	↓ Osteoblast apoptosis, ↑ Cell viability, ↑ Osteogenic differentiation, ↑ Matrix mineralization	[87]
YS-51S	HO-1-dependent inhibition of NF-κB activation	ROS 17/2.8 osteoblast-like cells	↓ NO production, ↓ Inflammatory responses	[88]
Z-Guggulsterone	Nrf2-mediated HO-1 induction and cytoprotection	Dex-treated MC3T3-E1 cells GIO rats	↓ Oxidative stress, ↓ Osteoblast injury, ↑ Osteogenic differentiation ↑ BMD, ↓ Bone loss, ↑ Bone microarchitecture	[89]

Note: ↑, increased; ↓, decreased.

**Table 2:** Pharmaceuticals and some synthetic formulations capable of modulating bone metabolism.

Agent	HO-1-Related Mechanism	Experimental Model	Biological Effects	[Ref.]
Existing drugs				
5-Aminolevulinic acid + sodium ferrous citrate	Activation of the Bach1/Nrf2/HO-1 pathway through promotion of Bach1 nuclear export	RAW264.7 cells PMs LPS-induced calvarial bone destruction mice	↓ Osteoclast differentiation ↓ Osteoclast differentiation ↓ Bone destruction	[90]
Dimethyl fumarate	Activation of Nrf2/HO-1 signaling with suppression of ROS-mediated osteoclastogenesis	RAW264.7 cells LPS-induced calvarial bone destruction mice	↓ Osteoclast differentiation, ↓ Bone resorption ↓ Inflammatory bone destruction	[91]
Melatonin	Activation of Nrf2/HO-1 signaling	MC3T3-E1 cells T2DOP rats	↑ Osteogenic differentiation ↑ Bone formation, ↑ Bone microarchitecture	[92]
Simvastatin	Enhancement of HO-1-mediated antioxidant defense	H_2_O_2_-treated MG-63 osteoblast-like cells Aged rats OVX rats	↓ Oxidative stress, ↓ Osteoblast apoptosis, ↑ ALP activity ↑ BMD ↑ BMD	[93]
SRT3025	FoxO-dependent induction of HO-1 following SIRT1 activation	BMMs	↓ Osteoclast differentiation	[94]
Emerging HO-1-targeting therapeutics				
CORM-2	CO-mediated suppression of ROS-dependent NF-κB/NFATc1 signaling downstream of HO-1	RAW264.7 cells BMMs	↓ Osteoclast differentiation ↓ Osteoclast differentiation, ↓ Bone resorption	[61]
CORM-3	CO-mediated suppression of inflammatory signaling downstream of HO-1	OVX + CIA mice	↓ Joint inflammation, ↓ Osteoclast surface, ↓ Bone erosion, ↑ Trabecular bone preservation	[95]
	Activation of the Nrf2/HO-1 pathway	H_2_O_2_-treated rat BMSCs Mouse BMMs OVX rats	↓ ROS, ↓ Mitochondrial dysfunction, ↓ Osteoblast apoptosis, ↑ Osteogenic differentiation ↓ Osteoclast differentiation ↑ Bone formation, ↓ Bone loss, ↑ BV/TV, ↑ Tb.N, ↓ Tb.Sp	[96]
4-Octyl itaconate	Suppression of Hrd1-mediated Nrf2 ubiquitination with activation of Nrf2/HO-1 signaling	BMMs LPS-stimulated BMMs OVX mice	↓ Osteoclast differentiation, ↓ F-actin ring formation, ↓ Bone resorption ↓ ROS production, ↓ Inflammatory responses ↓ Bone loss, ↓ Osteoclast number, ↑ BMD, ↑ BV/TV, ↑ Tb.Th	[97]
RTA-408 (Omaveloxolone)	Activation of Nrf2/HO-1 signaling with suppression of STING-dependent NF-κB signaling	BMMs OVX mice	↓ Osteoclast differentiation, ↓ Bone resorption, ↓ F-actin ring formation ↓ Bone loss, ↓ Osteoclast number, ↑ BV/TV, ↑ Tb.N, ↑ Tb.Th, ↓ Tb.Sp	[98]

Note: ↑, increased; ↓, decreased. BV/TV, bone volume/tissue volume; Tb.N, trabecular number; Tb.Sp, trabecular separation; BMD, bone mineral density; Tb.Th, trabecular thickness.

#### 4.2.3. Existing Drugs

Several existing pharmacological agents have demonstrated bone-protective effects through modulation of the HO-1 pathway (Table 2). Simvastatin potentiates HO-1-mediated antioxidant defenses, thereby reducing oxidative stress and osteoblast apoptosis while preserving osteoblast viability [93]. Similarly, melatonin promotes osteogenic differentiation through activation of Nrf2/HO-1 signaling and improves bone formation in experimental diabetic osteoporosis [92].

In addition to their osteogenic effects, other existing drugs predominantly suppress osteoclastogenesis and inflammatory bone destruction. Dimethyl fumarate suppresses ROS-dependent osteoclast differentiation through activation of Nrf2/HO-1 signaling, whereby it protects against inflammatory bone destruction [91]. 5-Aminolevulinic acid plus sodium ferrous citrate activates the Bach1/Nrf2/HO-1 pathway by promoting Bach1 nuclear export, resulting in marked suppression of osteoclastogenesis and inflammatory bone destruction [90].

#### 4.2.4. Emerging HO-1-Targeting Therapeutics

Recent advances in bone research have identified several emerging pharmacological strategies that target the Nrf2/HO-1 pathway or its downstream effectors to prevent pathological bone loss (Table 2). Use of carbon monoxide-releasing molecules (CORMs) represents an alternative strategy for modulating the HO-1 pathway through controlled delivery of CO, a major by-product of HO-1 activity. CORM-2 suppresses osteoclast differentiation through inhibition of ROS-dependent NF-κB/NFATc1 signaling [61], whereas CORM-3 attenuates oxidative stress-induced bone loss via the Nrf2/HO-1 pathway [96] and suppresses inflammatory signaling, resulting in reduced joint inflammation, osteoclast surface, and bone erosion, together with preservation of trabecular bone [95].

In addition, small-molecule activators of the Nrf2/HO-1 pathway have shown promising effects in experimental models of bone loss. 4-Octyl itaconate activates Nrf2/HO-1 signaling by suppressing Hrd1-mediated Nrf2 ubiquitination [97]. The Nrf2 activator RTA-408 (omaveloxolone) enhances Nrf2/HO-1 signaling while suppressing STING-dependent NF-κB activation [98]. Both compounds reduce oxidative stress, inhibit osteoclast formation and bone resorption, and preserve bone mass and trabecular microarchitecture in vivo [97,98].

### 4.3. Limitations and Future Perspectives

Despite growing evidence supporting the role of HO-1 in bone remodeling, several challenges remain before these findings can be translated into clinical practice. Most available evidence is derived from cell-based experiments and animal models, which cannot fully reflect the complexity of human bone diseases. In addition, many HO-1-modulating agents exert pleiotropic effects through multiple signaling pathways, making it difficult to distinguish non-canonical mechanisms from broader antioxidant or anti-inflammatory actions.

Another important consideration is the context-dependent nature of HO-1 signaling. While moderate HO-1 expression generally protects osteoblasts and suppresses osteoclastogenesis, excessive or dysregulated activation of HO-1-mediated signaling may contribute to ferroptosis under certain pathological conditions, particularly diabetic bone disease [41]. This apparent duality may arise from differences in disease context, cellular environment, and the magnitude and duration of HO-1 induction. Thus, HO-1 induction should not necessarily be regarded as universally beneficial, and systemic modulation may produce unintended effects when its cytoprotective and iron-related functions become imbalanced. Precision targeting of HO-1 also faces practical challenges. Most current inducers act systemically and lack bone-specific delivery, raising the risk of off-target effects. Achieving cell-type specificity within bone is equally difficult, given the opposing consequences of HO-1 modulation in osteoblasts, osteoclasts, and osteocytes [99]. Future studies should therefore focus on defining the optimal therapeutic window, improving bone-specific delivery strategies, and validating HO-1-targeted therapies in well-designed preclinical and clinical studies. Further investigation into the roles of HO-1 in ferroptosis, immunometabolism, osteocyte biology, and cellular senescence will also advance the development of more precise therapeutic approaches.

## 5. Conclusions

HO-1 is an important regulator of bone remodeling that integrates antioxidant, anti-inflammatory, and cytoprotective signaling to maintain the balance between bone formation and bone resorption. Through modulation of multiple signaling pathways, including those mediated by Nrf2, NF-κB, MAPK, and RANKL/RANK/NFATc1, HO-1 promotes osteoblast functions while limiting osteoclast differentiation and inflammatory bone destruction. Accumulating evidence supports HO-1 as a promising therapeutic target for osteoporosis and other bone disorders. However, the context-dependent effects of HO-1, particularly its dual role in ferroptosis, highlight the need for precise therapeutic modulation. Continued mechanistic studies together with advances in targeted drug delivery and clinical validation will be essential for translating HO-1-based strategies into effective therapies for skeletal diseases.

## Data Availability

No new data were created or analyzed in this study. Data sharing is not applicable to this article.

## Abbreviations

The following abbreviations are used in this manuscript:

ALP	Alkaline phosphatase
ARE	Antioxidant response element
ASCs	Adipose-derived stem cells
Bach1	BTB and CNC homology 1
BMD	Bone mineral density
BMMs	Bone marrow-derived macrophages
BMP-2	Bone morphogenetic protein-2
BMSCs	Bone marrow stromal cells
BMU	Basic multicellular unit
BV/TV	Bone volume/tissue volume
CIA	Collagen-induced arthritis
CO	Carbon monoxide
Col1a1	Collagen type I alpha 1
CoPP	Cobalt protoporphyrin IX
CORM-2	Carbon monoxide-releasing molecule-2
CORM-3	Carbon monoxide-releasing molecule-3
CORMs	Carbon monoxide-releasing molecules
CRNDE	Colorectal neoplasia differentially expressed
Dex	Dexamethasone
Dlx5	Distal-less homeobox 5
ERK	Extracellular signal-regulated kinase
Fe^2+^	Ferrous iron
FoxO	Forkhead box O
FTH1	Ferritin heavy chain
GIO	Glucocorticoid-induced osteoporosis
GPX4	Glutathione peroxidase 4
H_2_O_2_	Hydrogen peroxide
HIF-1α	Hypoxia-inducible factor 1-alpha
HMGB1	High mobility group box 1
HMOX1	Heme oxygenase-1 gene
HO-1	Heme oxygenase-1
IKK	Inhibitor of κB kinase
IL-1β	Interleukin-1β
IL-6	Interleukin-6
Keap1	Kelch-like ECH-associated protein 1
KLF7	Krüppel-like factor 7
lncRNAs	Long non-coding RNAs
LPS	Lipopolysaccharide
MAPK	Mitogen-activated protein kinase
MMP-9	Matrix metalloproteinase-9
MSCs	Mesenchymal stem cells
NFATc1	Nuclear factor of activated T cells 1
NF-κB	Nuclear factor kappa B
NO	Nitric oxide
NQO1	NAD(P)H quinone oxidoreductase 1
Nrf2	Nuclear factor erythroid 2-related factor 2
OC	Osteocalcin
OPG	Osteoprotegerin
OVX	Ovariectomized
PMs	Peritoneal macrophages
RANK	Receptor activator of nuclear factor kappa-B
RANKL	Receptor activator of nuclear factor kappa-B ligand
RNS	Reactive nitrogen species
ROS	Reactive oxygen species
Runx2	Runt-related transcription factor 2
SIRT1	Sirtuin 1
SnPP	Tin protoporphyrin
SOST	Sclerostin
STING	Stimulator of interferon genes
T2DOP	Type 2 diabetic osteoporosis
Tb.N	Trabecular number
Tb.Sp	Trabecular separation
Tb.Th	Trabecular thickness
TNF-α	Tumor necrosis factor-α
TRAF6	Tumor necrosis factor receptor-associated factor 6
TRAP	Tartrate-resistant acid phosphatase
VEGF	Vascular endothelial growth factor
